# Integration of anti-PD-1 antibody into chemotherapeutic regimens improved the outcome of aggressive NK cell leukemia: a single-center retrospective real-world analysis

**DOI:** 10.3389/fimmu.2025.1576904

**Published:** 2025-04-14

**Authors:** Kai Shen, Yi Liao, Yang Dai, Jie Ji, Pu Kuang, Zhigang Liu, Liping Xie, Ting Niu, Nenggang Jiang, Hongbing Ma

**Affiliations:** ^1^ Department of Hematology, Institute of Hematology, West China Hospital, Sichuan University, Chengdu, China; ^2^ Department of Laboratory Medicine, West China Hospital, Sichuan University, Chengdu, China

**Keywords:** aggressive natural killer cell leukemia, programmed cell death protein 1, immunotherapy, asparaginase, hematopoietic stem transplantation, prognosis

## Abstract

**Background:**

Aggressive natural killer (NK) cell leukemia (ANKL) is a rare NK cell neoplasm associated with Epstein-Barr virus (EBV) infection. Programmed cell death protein 1 (PD-1) blockade, which is successful in extranodal NK/T-cell lymphoma and EBV-related hemophagocytic lymphohistiocytosis, is considered to have a potential role in managing ANKL.

**Objectives:**

This study aims to characterize ANKL clinically and evaluate the prognostic impact of anti-PD-1 antibody treatment.

**Methods:**

We retrospectively analyzed the clinical characteristics and treatment regimens of ANKL patients from March 2009 to October 2023 in a single center. Data on clinical characteristics, treatment regimens and prognosis were collected from medical records. Overall survival (OS) of different risk groups was analyzed by Kaplan-Meier method. The least absolute shrinkage and selection operator (LASSO)-penalized Cox regression was used to identify the potential prognostic factors of ANKL.

**Results:**

From March 2009 to October 2023 a total of 71 ANKL were retrieved with an OS of 2.0 months. Seven patients (9.9%) received PD-1 antibodies combined with various chemotherapies; thirty-five patients (49.3%) received asparaginase as part of chemotherapy; and eight patients (11.3%) received allogeneic HSCT after induction chemotherapy. Among patients who did not undergo allogeneic hematopoietic stem transplantation (HSCT), patients who received PD-1 antibodies as part of chemotherapy exhibited a superior OS than those without PD-1 antibodies (5.4 vs 1.6 months, p=0.035). The 1-year OS rate was 43% in the PD-1 subgroup compared with only 4% in the non-PD-1 subgroup. LASSO-Cox multivariate analysis revealed that PD-1 antibodies-containing regimens were associated with better survival (hazard ratio [HR]=0.349, 95% CI: 0.145~0.840, p=0.019). So was it with HSCT and asparaginase (HR=0.267, 95% CI=0.101~0.701 and HR=0.355, 95% CI=0.206~0.613, respectively).

**Conclusion:**

ANKL still had a poor outcome in the past decade. Integration of anti-PD-1 antibody into chemotherapeutic therapy significantly improved the survival of ANKL. The prolonged survival attributed to PD-1 blockade could provide critical opportunities for patients awaiting HSCT.

## 1 Introduction

Aggressive natural killer (NK) cell leukemia (ANKL) is a rare hematological malignancy characterized by the Epstein-Barr virus (EBV)-driven proliferation of mature natural killer (NK) cells (1). ANKL accounts for only 0.1% of lymphoid neoplasms and most frequently affects younger patients between the ages of 20 and 30 predominantly in Eastern Asian countries (2). ANKL has a fulminant clinical course, with a median overall survival (OS) of less than 2 months (3). Life-threatening complications such as hemophagocytic lymphohistocytosis (HLH), resulting in high fever, splenohepatomegly, disseminated intravascular coagulopathy (DIC) and multiple organ failure (4).

Unfortunately, early attempts utilizing chemotherapeutic regimens of non-Hodgkin lymphomas have all ended with a dismal outcome in ANKL (5). The only potential cure is induction with L-asparaginase-based chemotherapy followed with allogeneic hematopoietic stem transplant (HSCT) (6). However, most patients with ANKL will not have the chance of transplantation because of its aggressive course, limited response to the current treatment and the unavailability of matched donors. In recent years, targeted drugs such as programmed cell death protein 1 (PD-1) monoclonal antibodies have been used to treat mature NK/T cell lymphoma with remarkable success (7, 8). Our center first reported the successful treatment of relapsed/refractory EBV-associated HLH (EBV-HLH) with PD-1 antibodies in 2020 (9). Considering the close bond between EBV and ANKL, PD-1 antibodies have also been tested in ANKL patients and may prolong the survival in a limited number of patients (10). Since the clinical evidence regarding its management predominantly comes from case reports and small-sample retrospective studies, there is yet no consensus on the treatment of ANKL.

In this study, we retrospectively analyzed the clinical characteristics and treatment regimens of ANKL patients in our single center to explore the prognosis of ANKL patients in a real-world setting and the impact of PD-1 antibodies on prognosis.

## 2 Methods

### 2.1 Study design and participants

This was a single-center retrospective analysis of ANKL patients who were diagnosed and treated at West China Hospital, Sichuan University (WCHSCU), from March 2009 to October 2023. The diagnosis of ANKL was adopted from the 2022 World Health Organization classification of lymphoid neoplasms (1), which included (1) the infiltration of atypical medium- to large-sized lymphocytes in the peripheral blood (PB), bone marrow (BM) or affected tissues; and (2) abnormal NK cells confirmed by flow cytometry (FCM) or immunohistochemistry (IHC). The exclusion criteria were as follows: (1) any other T or T-NK cell neoplasms confirmed by pathology, such as extranodal NK/T cell lymphoma (ENKTL), hepatosplenic and cutaneous γδT-cell lymphoma and peripheral T-cell lymphomas; (2) leukemic cells expressing clonal TCR confirmed by FCM or molecular pathological assays; and (3) pediatric patients aged younger than 14 years. Clinical data, including demographic information, clinical manifestations, laboratory parameters and outcomes, were collected from the electronic medical records. The follow-up period ended on December 14, 2023. This study was performed in accordance with the Declaration of Helsinki and approved by the Ethical Committee of WCHSCU.

### 2.2 Treatment

All ANKL patients with HLH were initially treated with the HLH-1994 protocol which was the most used approach for HLH. Chemotherapy was administered once the diagnosis of ANKL was established. In addition to the standard LVD protocol (pegasparaginase + vincristine + dexamethasone), chemotherapeutic regimens such as anthracycline-based regimens (CHOP or CHOPE) and gemcitabine-based regimens (GED, GEMOX, GDP or GLIDE) could also be used with or without asparaginase. Two kinds of asparaginase, L-asparaginase and pegasparaginase, were both allowed. The choice of the chemotherapy protocol was determined at the discretion of the attending physicians based on their experience and each ANKL patient’s individual condition. PD-1 antibodies (either nivolumab or sintilimab) have been integrated into chemotherapy for certain EBV-infected ANKL patients. Generally, anti-PD-1 antibody with a dose of 200mg was given on day 1 of each chemotherapeutic cycle. HSCT was performed for eligible patients with controlled disease after chemotherapy once a matched or haplo-matched donor was available. For patients who responded to anti-PD-1 antibody -containing chemotherapy and did not undergo HSCT, maintenance with the administration of anti-PD-1 antibodies every 3 to 4 weeks similar to the maintenance therapy for Hodgkin lymphoma was continued until disease progression.

### 2.3 Statistical analyses

All the statistical analyzes were performed with R version 4.4.3 (The R Foundation for Statistical Computing) and SPSS version 29 (IBM Corp., Armonk, NY, USA). Continuous variables are expressed as the median (range). Categorical variables are presented as frequencies and percentages. The OS was defined as the time from admission to death or the last follow-up and was calculated via the Kaplan–Meier method. OS was compared between each risk group via the log-rank test. In the first-round multivariate analysis, variables with p values < 0.15 in the univariate analysis were firstly included using the forward stepwise Cox proportional hazard model. Secondly, a least absolute shrinkage and selection operator (LASSO) method was used to select and minimize prognostic variables using the R package “glmnet”. The LASSO-selected variables were further inputted into Cox analysis to determine the final results. A two-tailed p value < 0.05 was regarded as statistically significant.

## 3 Results

### 3.1 Baseline characteristics

**Figure 1:**
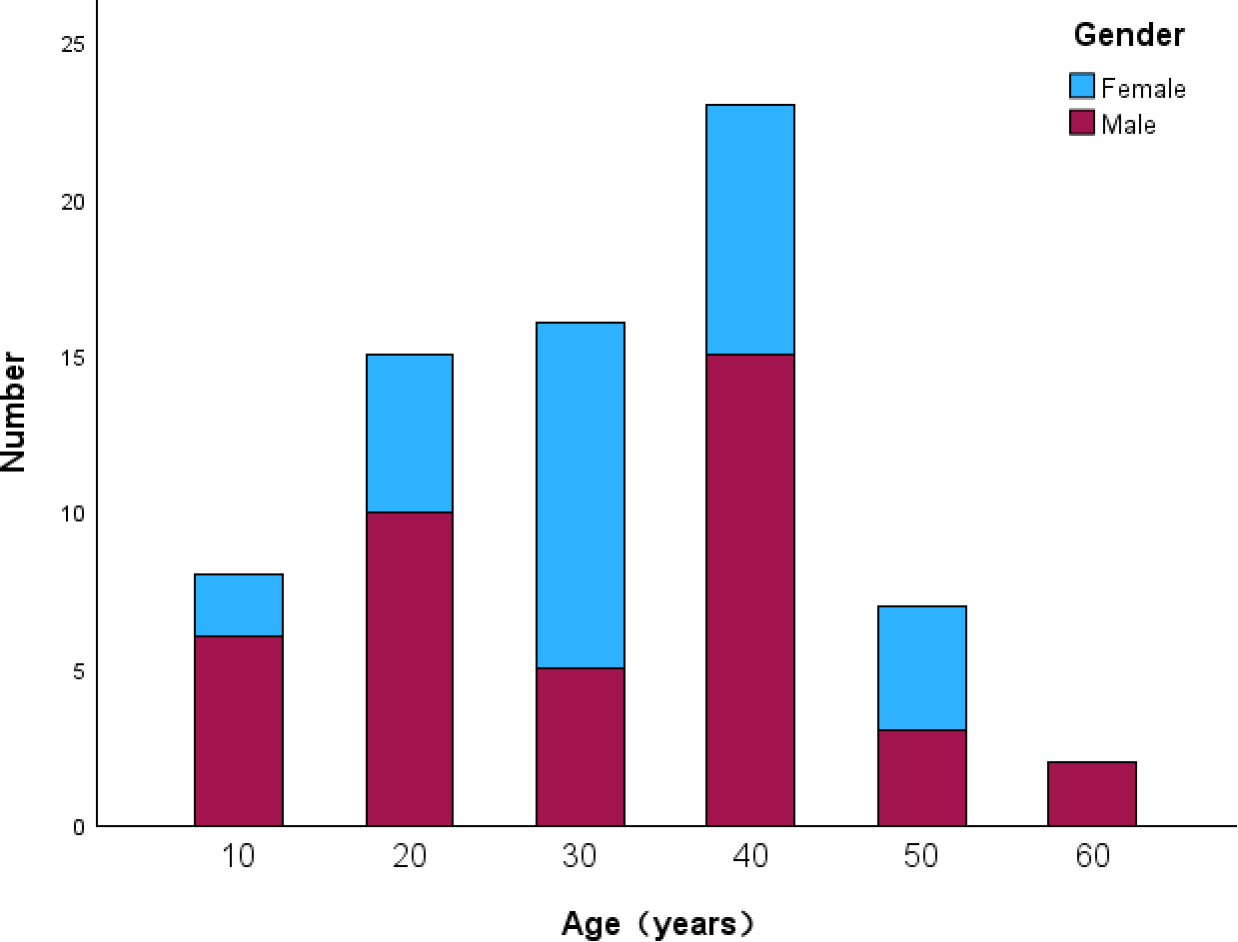
Age and sex distribution of the ANKL patients.

A total of 71 patients diagnosed with ANKL were treated in our center from 2009 to 2023. Forty-one (57.7%) patients were male, and the male-to-female ratio was 4:3. The median age was 37 years (range, 14–65 years) (**Figure 1**). The most common presentations were fever (95.8%) and splenomegaly (78.9%). Fifty-five (77.5%) patients had HLH at diagnosis. Eleven (15.5%) patients manifested infectious mononucleosis (IM)-like symptoms (including fever, lymphocytosis or mononucleosis, lymphadenopathy, and hepatosplenomegaly) for more than 90 days prior to the fulminant course of ANKL, and they were categorized as having “subacute ANKL”. For all patients, the median time from onset to diagnosis was 46 days (range, 10–2425 days). Among them, eight patients were previously diagnosed with chronic EBV infection (CAEBV). The peripheral blood EBV-DNA load was assayed in 59 patients, and a positive quantification result was found in 56/59 (94.9%) patients.

**Figure 2:**
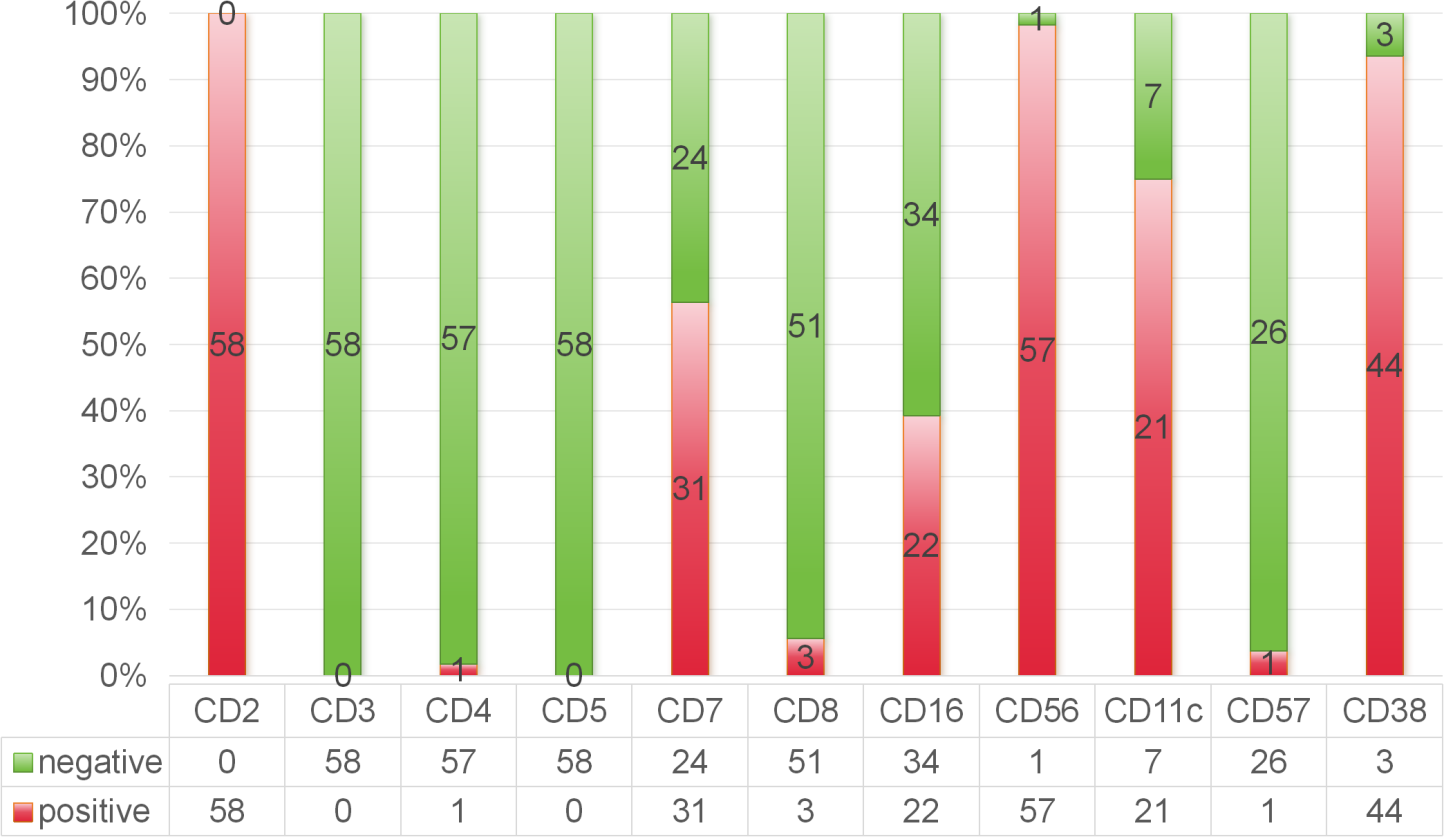
Immunophenotyping feature of ANKL by flow cytometry analysis of the bone marrow aspirates.

Immunophenotyping via FCM was conducted on the BM aspirates of 58 patients. The median percentage of abnormal NK cells was 8.4% (range, 0.2–96%). The percentage of abnormal cells was no more than 28.5% in 75% of the assayed cases. Most patients were positive for CD2 (100%), CD56 (98%), CD38 (94%) and CD11c (75%) and negative for surface CD3 (0%), CD4 (2%), CD57 (4%) and CD8 (6%). Fifty-six percent of the patients were positive for CD7, and 39% were positive for CD16 (**Figure 2**). Eight patients (9.9%) received congenital HLH-related genetic tests, and four patients had positive heterozygous mutations involving the PRF, SLC7A7, LYST, and UNC13D genes. The baseline clinical and laboratory characteristics are summarized in **Table 1**.

**Table 1:** Clinical characteristics of the ANKL patients.

Characteristics	Total patients (n=71)
	N (%)/Median (range)
Male	41 (57.7)
Female	30 (42.3)
Age (years)	37 (14~65)
Fever	68 (95.8)
Splenomegaly	56 (78.9)
Hepatomegaly	13 (18.1)
Onset to hospitalization (days)	34 (7 ~1801)
Subacute clinical onset	11 (15.5%)
Presence of HLH	55 (77.5)
Hemoglobin (g/L)	96 (10~159)
Platelet (×10^9/L)	42 (3~367)
WBC (×10^9/L)	2.05 (0.11~10.92)
Direct bilirubin (mmol/L)	7.65 (2~137.9)
ALT (U/L)	80 (10~719)
ALP (U/L)	203 (31~1103)
LDH (U/L)	667.5 (90~11453)
Triglyceride	2.72 (0.87~8.60)
Fibrinogen (g/L)	1.5 (0.4~7.7)
Serum creatinine (μmol/L)	58 (23~200)
EBV-DNA load (×10^3/L)	84 (0.12~15400)
Serum ferritin (ng/ml)	3390 (189 ~32000)

HLH, hemaphagocytic lymphohistiocytosis; WBC, white blood cell; ALT, alanine aminotransferase; ALP, alkaline phosphatase; LDH, lactate dehydrogenase; EBV-DNA, Epstein-Barr virus deoxyribonucleic acid.

### 3.2 Treatment choices

In terms of treatment, seven patients (9.9%) received PD-1 antibodies combined with various chemotherapies; thirty-five patients (49.3%) received asparaginase as part of chemotherapy; and eight patients (11.3%) received allogeneic HSCT after induction chemotherapy. None of the eight HSCT patients used PD-1 antibodies in the previous chemotherapy period prior to HSCT. The clinical details of the patients who received PD-1 therapy are summarized in **Table 2**. Notably, for patients presented with active HLH, ED regimen from HLH-1994 protocol was used to control the HLH prior to the initiation of anti-PD-1 antibody treatment.

**Table 2:** Detailed description of ANKL patients using PD-1 antibody.

No.	Age /Sex	Presentation	Hb (g/L)	Plt (×10^9/L)	WBC (×10^9/L)	ALT (U/L)	LDH (U/L)	Fib (g/L)	Ferritin (ng/L)	EBV-DNA (copies/L)	Abnormal NK cell by FCM	CD56	CD16	Hemophagocytosis in the BM	CAEBV history	Chemotherapy regimens	Cause of death	OS (months)
1	22/F	fever, lymphadenopathy and splenomegaly	68	184	0.98	396	1003	1.52	545.2	72,400	40.0%	bright	bright	yes	no	ED -> GED+PD-1	sepsis	2.2
2	31/F	fever, jaundice, splenomegaly and hepatomegaly	104	133	4.50	120	424	1.40	850	1,500,000	54.5%	bright	dim	no	no	LVD -> LVD+PD-1	disease progression	2.8
3	34/F	fever and splenomegaly	47	3	0.22	214	1564	0.40	8871	15,400,000	0.5%	bright	dim	yes	no	ED -> GLIDE+PD-1 ->PD-1	disease relapse	32.0
4	43/M	fever and splenomegaly	91	13	3.92	26	798	1.68	1040	588,000	1.1%	bright	dim	no	no	GLIDE -> LVD+PD-1	disease progression	5.4
5	15/F	fever and splenomegaly	55	19	0.47	39	1488	0.97	21843	3,940	6.9%	bright	bright	no	no	GLIDE+PD-1 -> PD-1	disease relapse	44.3
6	26/F	fever, jaundice, splenomegaly and hepatomegaly	60	16	0.92	102	5721	0.50	22418	7,450,000	7.0%	bright	dim	yes	yes	ED -> ED+PD-1 -> PD-1	disease relapse	24.1
7	42/F	fever, splenomegaly and CNS involvement	95	83	9.11	45	706	1.17	2518	17,200	4.0%	bright	dim	no	no	GLIDE+PD-1	disease progression	0.7

F, female; M, male; Hb, hemoglobin; Plt, platelet; WBC, white blood cell; ALT, alanine aminotransferase; LDH, lactate dehydrogenase; Fib, fibrinogen; EBV-DNA, Epstein-Barr virus deoxyribonucleic acid; FCM, flow cytometry; CAEBV, chronic active Eptsein-Barr virus infection. ED, etoposide and dexamethasone; GED, gemcitabine, etoposide and dexamethasone; PD-1, programmed cell death protein 1; LVD, l-asparaginase, etoposide and dexamethasone; GLIDE, gemcitabine, l-asparaginase, ifosfamide, dexamethasone and etoposide; CNS, central nervous system.

### 3.3 Survival and risk factors

**Figure 3:**
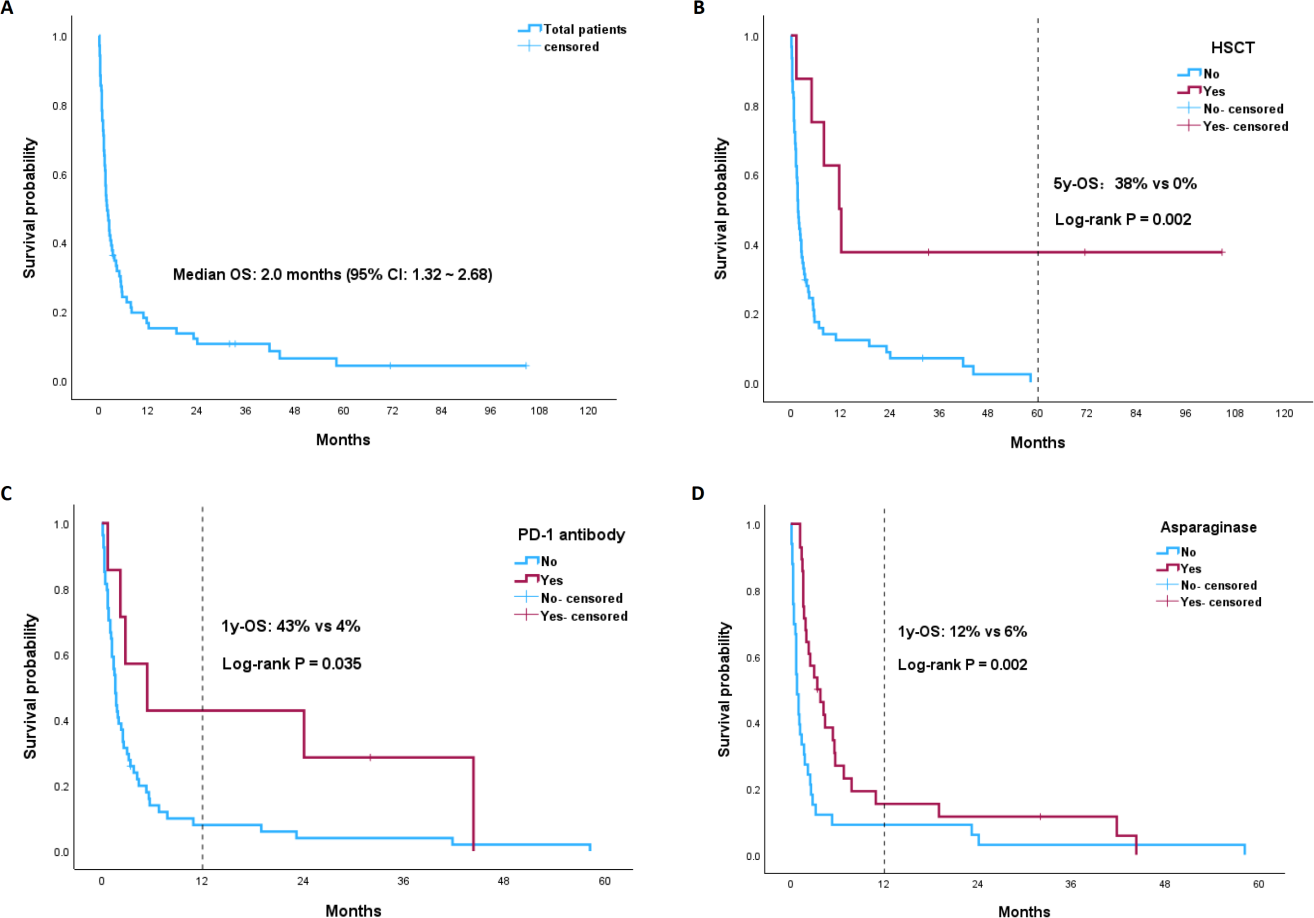
Kaplan-Meier (KM) plots of OS stratified by different risk groups: **(A)** the total ANKL patients; **(B)** HSCT vs non-HSCT group; **(C)** PD-1 antibody vs non-PD-1 antibody subgroup among the non-HSCT ANKL patients; **(D)** asparaginase vs non-asparaginase subgroup among the non-HSCT ANKL patients.

At a median follow-up of 71.4 months (95% confidence interval [CI]: 15.73~127.07), the median OS of the total patients was 2.0 months (95% CI: 1.32~2.68) (**Figure 3A**). The 1-year OS rate was 15%. Patients who underwent HSCT had significantly longer OS than non-HSCT patients (11.7 vs 1.7 months, p=0.002) (**Figure 3B**). The 5-year OS rate of HSCT patients was 38%, whereas no patients without HSCT survived 5 years. As none of the eight HSCT patients received PD-1 antibody immunotherapy during induction chemotherapy, we further analyzed the impact of the PD-1 antibody on the survival of the non-HSCT group. Among the patients who did not undergo HSCT, patients who received PD-1 antibodies had a longer OS than did those who did not receive PD-1 antibodies (5.4 vs 1.6 months, p=0.035) (**Figure 3C**). The 1-year and 2-year OS rates for the PD-1 antibody subgroup were 43% and 26%, respectively, whereas the 1-year and 2-year OS rates for the non-PD-1 antibody subgroup were both as low as 4%. Similarly, non-HSCT patients treated with asparaginase-containing chemotherapy had longer OS than non-HSCT patients did (4.1 vs 1.1 months, p=0.002) (**Figure 3D**). The 1-year OS rate of the asparaginase subgroup was 12%, whereas for the non-asparaginase subgroup, the 1-year OS rate decreased to 6%.

A variety of clinical features and therapeutic strategies as possible prognostic factors were evaluated by univariate analysis for all patients (**Table 3**). In addition to HSCT (p=0.002), asparaginase was also shown to be associated with better OS (p <0.001). PD-1 antibody, subacute clinical course and the presence of HLH marginally affected OS (p<0.15) and were included in the multivariate analysis. Multivariate analysis revealed that both HSCT and asparaginase were associated with better OS (hazard ratio [HR]=0.237, 95% CI=0.092~0.613 and HR=0.45, 95% CI=0.272~0.746, respectively). Moreover, PD-1 antibodies were also demonstrated to be valuable predictors of survival (HR=0.406, 95% CI: 0.172~0.958). The survival advantage of a subacute clinical course, which was not statistically significant according to univariate analysis, was revealed by multivariate analysis (HR=0.446, 95% CI: 0.215~0.923) (**Table 4**).

**Table 3:** Univariate analysis of variables related to survival.

Variable		n	median OS	95% CI	*p*-Value
Gender	male	41	2.5	1.496 ~ 3.504	0.768
	female	30	1.4	0.864 ~ 1.936	
Age	≥37 years	39	1.9	1.324 ~ 2.676	0.630
	<37 years	32	2.1	1.324 ~ 2.876	
Splenomegaly	yes	56	2.1	1.275 ~ 2.925	0.378
	no	15	1.9	1.395 ~ 2.405	
Hepatomegaly	yes	13	2.8	0 ~ 8.319	0.229
	no	58	1.7	1.202 ~ 2.198	
Subacute clinical course	yes	11	4.2	0.000 ~ 8.408	0.142
	no	60	1.7	1.131 ~ 2.269	
HLH	yes	55	2.3	1.170 ~ 3.430	0.105
	no	16	1.6	0.032 ~ 3.168	
Hemoglobin	≥90g/L	44	2.1	1.369 ~ 2.831	0.771
	<90g/L	27	1.8	1.122 ~ 2.478	
Platelet	≥30×10^9/L	50	2.0	1.307 ~ 2.693	0.376
	<30×10^9/L	21	1.8	0.305 ~ 3.295	
WBC	≥2×10^9/L	48	1.9	1.288 ~ 2.512	0.982
	<2×10^9/L	42	2.5	0.975 ~ 4.025	
Direct bilirubin	≥20mmol/L	16	3.8	1.000 ~ 5.400	0.203
	<20mmol/L	55	1.8	1.272 ~ 2.328	
ALT	≥80mmol/L	12	2.5	1.550 ~ 3.450	0.666
	<80mmol/L	59	1.2	0.467 ~ 1.933	
ALP	≥200mmol/L	34	2.2	1.343 ~ 3.057	0.995
	<200mmol/L	37	1.7	1.104 ~ 2.296	
LDH	≥1000U/L	21	2.8	1.525 ~ 3.675	0.164
	<1000U/L	50	1.7	0.536 ~ 2.264	
Triglyceride	≥3mmol/L	32	1.6	1.046 ~ 2.154	0.326
	<3mmol/L	39	2.5	1.154 ~ 3.846	
Creatinine	≥90μmol/L	8	1.7	0.000 ~ 5.581	0.818
	<90μmol/L	63	2.0	1.319 ~ 2.681	
Fibrinogen	≥1.5g/L	36	2.1	1.394 ~ 2.806	0.577
	<1.5/L	35	1.7	0.773 ~ 2.627	
EBV-DNA	≥1×10^5copies/ml	10	2.2	0.000 ~ 5.425	0.884
	<1×10^5copies/ml	49	1.8	1.047 ~ 2.553	
Serum ferritin	≥3500ng/ml	36	2.0	0.871 ~ 2.929	0.960
	<3500ng/ml	35	1.9	0.938 ~ 3.062	
CD16^bright^	yes	22	3.7	0 ~ 6.610	0.289
	no	34	1.7	1.473 ~ 1.927	
HSCT	yes	8	11.7	5.879 ~17.521	0.002
	no	63	1.7	1.268 ~ 2.132	
PD-1 antibody	yes	7	5.4	0.000 ~ 12.072	0.140
	no	64	1.7	1.230 ~ 2.170	
Asparaginase	yes	35	4.4	2.181 ~ 6.619	<0.001
	no	36	1.0	0.530 ~ 1.470	

HLH, hemaphagocytic lymphohistiocytosis; WBC, white blood cell; ALT, alanine aminotransferase; ALP, alkaline phosphatase; LDH, lactate dehydrogenase; EBV-DNA, Epstein-Barr virus deoxyribonucleic acid; ALT, alanine aminotransferase; HSCT: hematopoietic stem cell transplantation; PD-1, programmed cell death protein 1.

**Table 4:** Risk factors of OS identified by the first-round multivariate Cox analysis.

Variables	Coefficient	Standard error	Walds	*p*-Value	HR	95% CI
PD-1 antibody: yes vs no	-0.901	0.438	4.237	0.040	0.406	0.172 ~ 0.958
Asparaginase: yes vs no	-0.798	0.258	9.609	0.002	0.450	0.272 ~ 0.746
HSCT: yes vs no	-1.44	0.485	8.822	0.003	0.237	0.092 ~ 0.613
Subacute clinical course: yes vs no	-0.808	0.371	4.733	0.030	0.446	0.215 ~ 0.923

PD-1, programmed cell death protein 1; HSCT, hematopoietic stem cell transplantation.

### 3.4 LASSO - penalized Cox analysis

**Figure 4:**
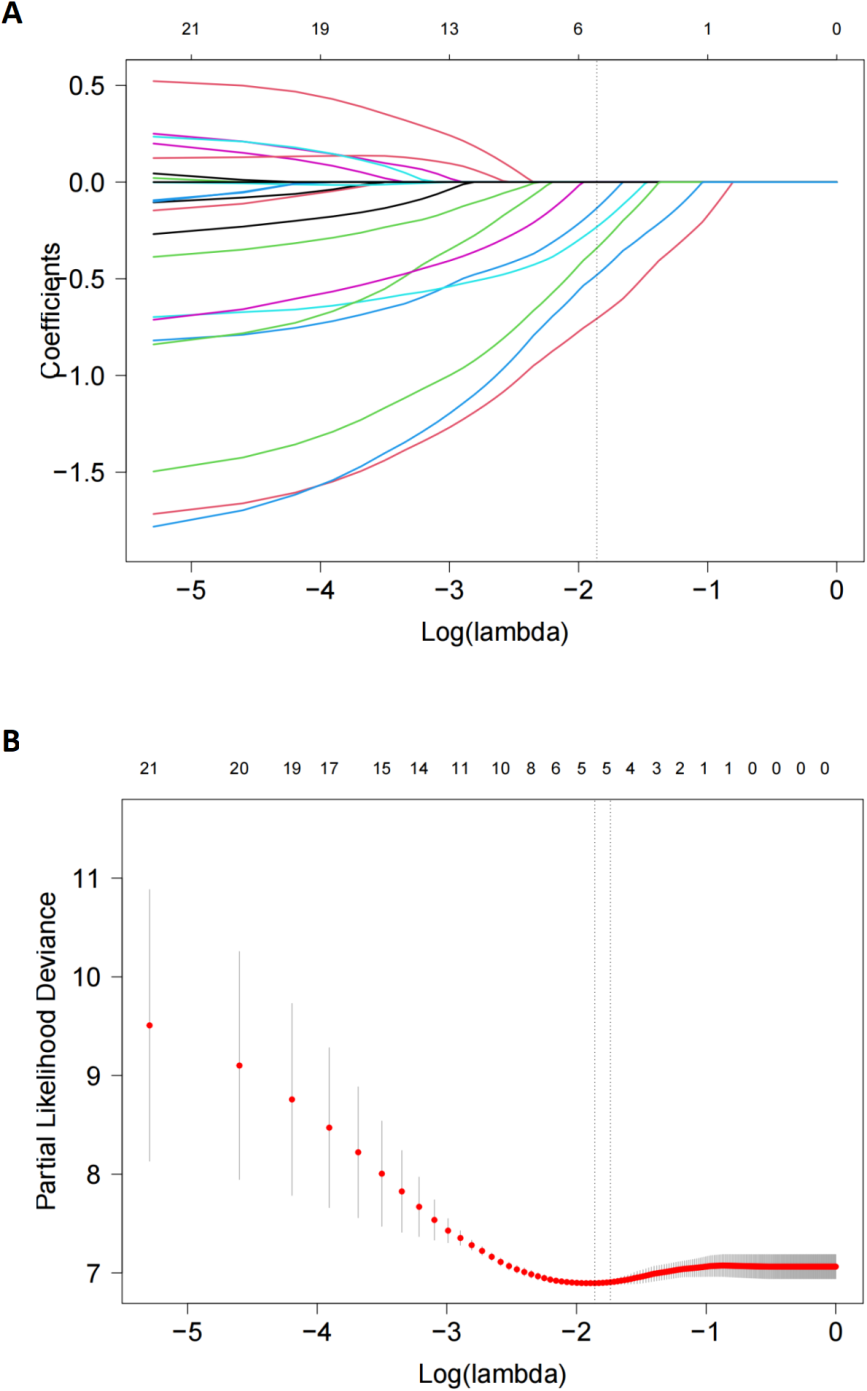
LASSO Cox regression model to select candidate variables associated with OS. **(A)** LASSO coefficient profiles of 21variables associated with OS. A dotted vertical line was drawn at the optimal λ value (0.176) chosen by 10-fold cross-validation. **(B)**. Five risk factors selected using LASSO Cox regression analysis. The two dotted vertical lines were drawn at the optimal scores which resulted in 5 nonzero coefficients (hepatomegaly,-0.056; subacute clinical course, -0.165; HSCT, -0.646; PD-1 antibodies, -0.259; asparaginase, -0.408).

Considering the small sample number of the entire cohort and the limited number of PD-1 antibody subgroup, LASSO model was used and identified five potential prognostic factors, including splenomegaly, subacute clinical course, HSCT, PD-1 antibodies and asparaginase (**Figure 4**). Cox regression model was established based on all factors screened by LASSO regression even though hepatomegaly had a small coefficient of 0.054 close to zero. This new cox model still confirmed that PD-1 antibody was a significant prognostic factor for OS (HR=0.345, 95% CI: 0.145~0.840, p=0.019) (**Table 5**). So was it with HSCT and asparaginase (HR=0.267, 95% CI=0.101~0.701 and HR=0.355, 95% CI=0.206~0.613, respectively). A nomogram was developed based on the selected predictors to provide a visual tool for OS risk assessment (**Supplementary Figure S1**). The resulting nomogram showed excellent discrimination with the area under the curve (AUC) of 0.800, 0.845, and 0.849 for 1-, 2-, and 3-year survival, respectively (**Supplementary Figure S2**). Meanwhile, the 1- year and 2- year calibration curves demonstrated that this nomogram had good forecast precision (**Supplementary Figure S3**).

**Table 5:** Risk factors of OS identified by LASSO-Cox regression model.

Variables	Coefficient	Standard error	Walds	*p*-Value	HR	95% CI
Hepatomegaly: yes vs no	-0.797	0.36	-2.216	0.027	0.451	0.223 - 0.912
Subacute clinical course: yes vs no	-0.763	0.365	-2.089	0.037	0.466	0.228 - 0.954
HSCT: yes vs no	-1.322	0.496	-2.668	0.008	0.267	0.101 - 0.704
PD-1 antibodies: yes vs no	-1.052	0.448	-2.35	0.019	0.349	0.145 - 0.840
Asparaginase: yes vs no	-1.034	0.278	-3.724	<0.001	0.355	0.206 - 0.613

PD-1, programmed cell death protein 1; HSCT, hematopoietic stem cell transplantation.

## 4 Discussion

In our study we explored the clinical features and prognostic predicators of ANKL, a highly aggressive subtype of NK-cell neoplasm, in a real-world setting. A sample size of 71 in a single center which was accumulated in a span of more than 10 years, was considered large if the rarity of ANKL was taken into consideration. The median age was 37 years, and the incidence peak was around the 30s and 40s. Like the multicenter retrospective study of ANKL in the Chinese Han ethnicity by Tang et al (3), a subgroup of ANKL patients with a subacute clinical course was also demonstrated in our study, accounting for 15.5% of the total patients. Although this subtype resembled CAEBV-transformed ANKL in clinical presentation, they could be distinguished in onset age, clinical course, and prognosis. ANKL transforming from CAEBV could be younger and might have a slightly better outcome. The multivariate analysis in our study also revealed better survival in patients with this subacute subtype of ANKL.

FCM is a valuable tool for differentiation between ANKL and other NK-cell neoplasms. Typical ANKL cells are shown to have a prominent forward scatter, which is consistent with a larger cell size, and the expression of CD56 (CD56bright) (11). In a report with 12 European patients, neoplastic cells appeared to consistently express CD2 with CD16 and the presence of CD16 in ANKL patients could distinguish the disease from ENKTL, which was usually CD16 negative (12). Nevertheless, in our study, CD16 positivity was only 39%. Lower expression of CD16 on FCM seemed to be much more common in Chinese and Japanese ANKL patients. In a previous report of 47 Chinese ANKL patients, 91.9% had a CD56bright/CD16dim- and CD57-negative phenotype (13).The CD56bright/CD16dim group could also represent an earlier stage of differentiation of either neoplastic or nonneoplastic NK cells (14). Thus, the diagnosis of ANKL should be cautious when an atypical immunophenotype was revealed by FCM, especially for those with a suspicious background of EBV-HLH or CAEBV. Another diagnostic challenge is the common presence of only few neoplastic NK cells in the BM at the early stage. The median percentage of abnormal NK cells in our study was 8.4%, similar to a previous report in which the proportion of abnormal NK cells in the BM was less than 5% in half of the patients (15). Therefore, repeated BM aspirations are often needed for the diagnosis.

L-asparaginase disrupted the asparagine supply to tumor cells and induced the apoptosis of ANKL neoplastic cells (16). Several clinical studies have shown that L-asparaginase-based regimens are associated with survival of ANKL. The overall response rates for patients treated with L-asparaginase-containing regimens were between 40% and 70%, so that eligible patients can be followed with HSCT (17, 18). Nevertheless, early referral and the initiation of donor search are recommended, as outcomes are poor without HSCT. In our study, the 5-year OS rate of the HSCT group was 38%, whereas there was no long-term survivor in the non-HSCT group. Unfortunately, previous studies have shown that fewer than one-third of patients are able to undergo HSCT (6, 19, 20). Moreover, long-term outcomes of HSCT remained dismal internationally, with a median OS of less than 1 year, primarily due to high disease relapse rate within the first year after HSCT (6, 20, 21). In our study, the OS of the HSCT patients was 11.7 months, which was consistent with previous reports. Thus, there is a large unmet need for more effective therapeutic agents for relapsed or refractory (R/R) ANKL or for those who are unable to tolerate intensified chemotherapy.

Recent studies have identified 4 major dysregulated pathways involved in ANKL pathogenesis: epigenetic modulation, TP53 and DNA repair, JAK/STAT/MYC, and the PD-L1/PD-1 checkpoint (11, 22). Because of the similarity between ENKTL and ANKL, PD-1 blockade was considered to have a potential role in managing ANKL (23). They may be particularly promising for EBV-positive ANKLs. Early research revealed that PD-L1 expression on EBV-transformed cancer cells was mediated by latent membrane protein 1 which could play a vital role in immune evasion (24). A pathological study found that approximately one-third of the ANKL cases overexpressed PD-L1 (7). Our colleague Liu P et al (9) first tested the PD-1 inhibitor nivolumab in 7 R/R EBV-HLH patients, and 5/7 patients achieved complete clinical remission, with a median follow-up of 16 months. Nivolumab expanded PD-1-positive T cells reserve and normalized cytotoxic T cell function by promoting the expression of degranulation genes and costimulatory genes. This normalization of the cytotoxic activation process was closely correlated with EBV clearance in in HLH. Inspired by their success and considering the association between ANKL and EBV infection, we integrated PD-1 monoclonal antibodies as immunotherapy into chemotherapy regimens as both induction and maintenance therapies for ANKL.

For patients with flaring HLH at diagnosis, a three-step strategy (cooling, consolidation, and reconstruction) was first proposed for EBV-associated lymphoproliferative disorders by Swada et al (25). Similarly, after ANKL patient’s hyperinflammatory condition was alleviated (or cooled) by HLH-2004-like chemotherapy, PD-1 antibodies could be integrated into the ensuing intensified chemotherapy (consolidation), such as the GLIDE regimen, which was designed for NK/T cell lymphoma by our colleagues Ji J et al (26). It was noteworthy that immunotherapy like PD-1 blockade can lead to the overactivation of immune system, resulting in cytokine storm and HLH (27). Thus, the initiation of anti-PD-1 antibody treatment in ANKL should be cautious with preexisting HLH and “cooling” with etoposide-based chemotherapy was the key to avoid disastrous HLH flares which could be manifested as a sudden worsening of HLH-related symptoms. Meanwhile, serial monitoring of HLH parameters like serum ferritin and soluble IL-2 receptor is important in the early recognition of this immunotherapy-related complication. In our study, the PD-1 antibody subgroup of non-HSCT patients presented a superior OS than the PD-1 antibody subgroup of non-HSCT patients (5.4 vs 1.6 months, p=0.035). Even though PD-1 antibodies cannot eventually prevent relapses, survival was obviously prolonged, as 3 out of the 7 PD-1 antibody-treated patients survived for more than 12 months. We believe that the prolongation of survival time attributed to PD-1 blockade could provide transplantation-eligible patients with a valuable window to search for suitable donors and increase the opportunity for a peaceful transition to HSCT by increasing the response rate as part of frontline chemotherapy and reducing early relapse as maintenance. Thus, in the future, when we gain more experience in using immune checkpoint inhibitors for ANKL, more work could be done, such as devising prospective studies integrating both PD-1 inhibitors and asparaginase into frontline and bridging chemotherapy for transplant-eligible patients. Moreover, PD-1 inhibitors might be used after HSCT as maintenance therapy to prevent relapses.

This study had certain limitations. Firstly, as a retrospective study at a single center, some clinical details like the PD-L1 expression level on pathology and serial EBV-DNA monitoring data could not be as intact as those in prospective trials, which could limit the number of predictors in the multivariate analysis. Secondly, owing to the rarity of the disease and the real-world setting, the patients enrolled in our study had a prolonged time span. Thus, era effect might be a confounding factor as data completeness and the timeliness of diagnosis was improving over time. Thirdly, treatment regimens in our study could only be broadly categorized. This heterogeneity in treatment protocols could lead to certain biases. Moreover, the small number of patients in PD-1 antibodies subgroup might limit the statistical strength even though a LASSO-Cox model was used. Therefore, future research should focus on well-designed multi-center prospective trials incorporating mechanistic investigations to further validate the results of this study.

## 5 Conclusion

ANKL, a rare but extremely aggressive NK cell neoplasm, still had a poor outcome in the past decade according to our single-center experience. Asparaginase-containing chemotherapy followed by HSCT was the only possible cure for ANKL. Most importantly, the integration of an anti-PD-1 antibody into induction chemotherapy and maintenance therapy could significantly improve the survival of ANKL patients. The prolonged survival attributed to PD-1 blockade could help transplantation-eligible patients gain more time to search for suitable donors and increase the opportunity for a peaceful transition to HSCT. In the future, regimens containing novel therapeutic agents with potential efficacy, including PD-1 antibodies, should be validated by multi-center prospective trials.

## Data Availability

The raw data supporting the conclusions of this article will be made available by the authors, without undue reservation.
