# Supplementary material for: Integration of anti-PD-1 antibody into chemotherapeutic regimens improved the outcome of aggressive NK cell leukemia: a single-center retrospective real-world analysis
Source: Front Immunol. 2025 Apr 14;16:1576904. doi: 10.3389/fimmu.2025.1576904 (PMC12034759; doi:10.3389/fimmu.2025.1576904)

Figure S1. Nomogram including the five key factors identified by LASSO-COX regression for the 1-year, 2-year and 3- year overall survival.


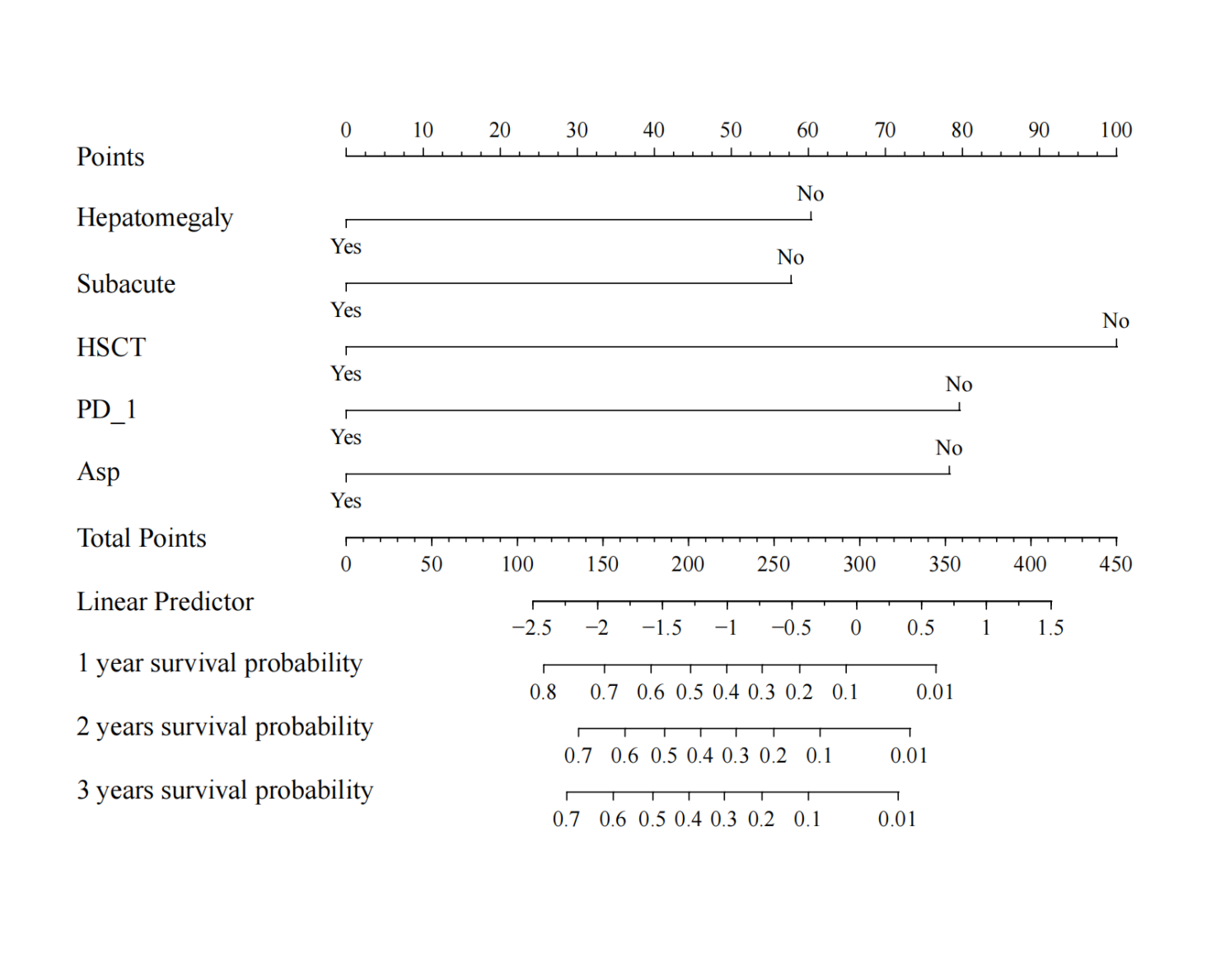


Figure S2. **ROC Analysis** demonstrated robust discriminatory power (AUC > 0.8 at all time points)


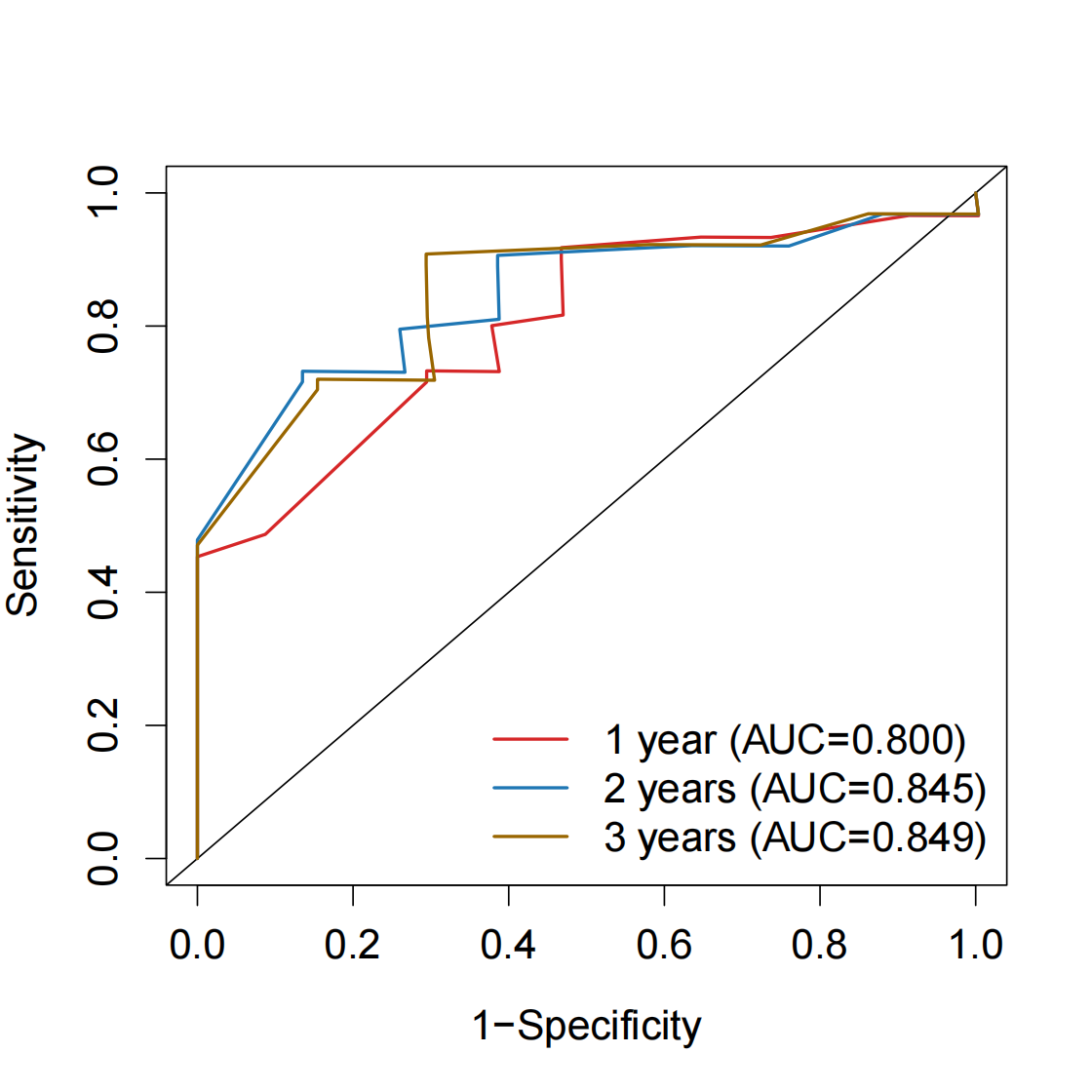


Figure S3. 1-year and 2-year calibration plots indicate a strong calibration.


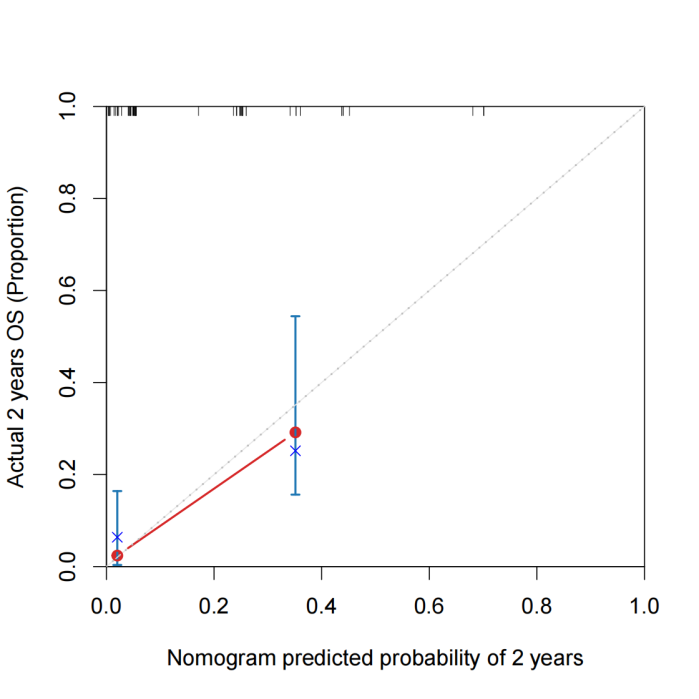

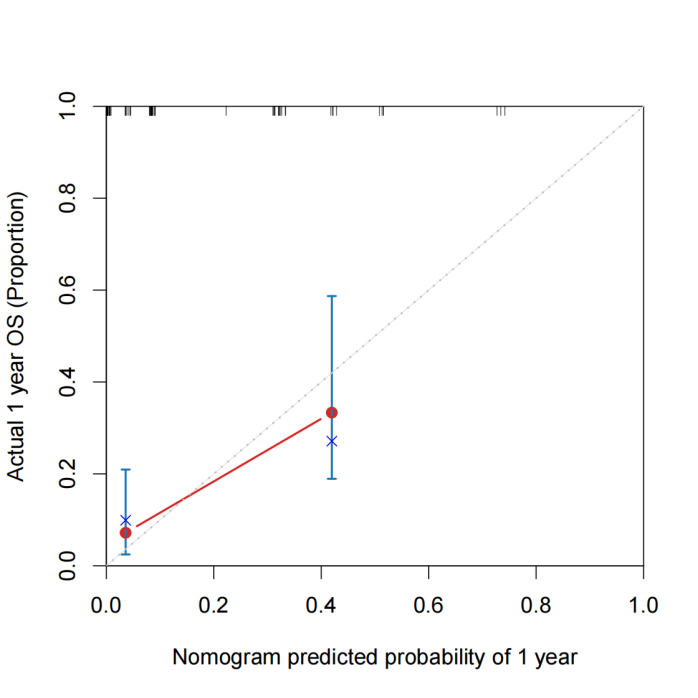

Supplement: Supplementary file 1 [file DataSheet1.docx]
